# Polystyrene nanoplastics promotes inflammation and aging in young mice through the oral-gut microbiome axis

**DOI:** 10.3389/fimmu.2026.1806158

**Published:** 2026-05-28

**Authors:** Ying Wang, Chunli Dong, Yi Xiao

**Affiliations:** 1 The First People’s Hospital of Zunyi, The Third Affiliated Hospital of Zunyi Medical University, Zunyi, Guizhou, China; 2 Institute of Life Sciences, Zunyi Medical University, Zunyi, Guizhou, China

**Keywords:** aging, inflammation, mice, oral-gut microbiome axis, polystyrene nanoplastics

## Abstract

With the escalating global pollution of nanoplastics, their impacts on organismal health have become a focal concern. The oral-gut microbiota axis plays a pivotal role in host health regulation, yet how nanoplastics influence this axis and drive inflammation and aging in young organisms remain undefined. This study aimed to investigate whether polystyrene nanoplastics (PS-NPs) promote inflammation and aging in young mice by disrupting the oral-gut microbiota axis. Therefore, we established a free-feeding model with 1000 μg/L PS-NPs using 8-week-old C57BL/6 mice. We quantified tissue inflammatory cytokines and cellular senescence markers to assess PS-NPs-induced inflammatory and aging effects, while 16S rRNA sequencing was employed to characterize oral and gut microbiota structural changes. We found that PS-NPs exposure significantly increased the expression levels of cellular senescence markers p21^Cip1/Waf^ and p16^Ink4a^ in lung and liver. Meanwhile, PS-NPs promoted the release of inflammatory cytokines such as IL-1β, IL-6 and TNF-α, by modulating the p38 MAPK pathway. In addition, PS-NPs also decreased the expression levels of antioxidant genes. Furthermore, 16S rRNA sequencing analysis revealed that PS-NPs exposure caused dysbiosis in oral and intestinal microbiota, manifested as significant alterations in microbial diversity and community structure. Our work provided mechanistic insights into nanoplastic toxicity and theoretical basis for developing preventive strategies.

## Introduction

Nanoplastics, defined as plastic particles with a size of less than 1000 nm, represent the final products of plastic degradation and are now widespread in water, soil, air, and various aspects of human life (1). They can enter the human body through multiple routes, including drinking water, food ingestion, and inhalation, gradually accumulate in organs such as the intestine, liver, and lungs over time, and thus pose a potential threat to human health (2, 3). What is more concerning is that the health risks of nanoplastics arise not only from their physical presence but also from their capacity to act as vectors. Their high specific surface area facilitates the adsorption of environmental contaminants such as heavy metals (e.g., copper ions), persistent organic pollutants (POPs), and pathogenic microorganisms, leading to combined pollution effects. One study has demonstrated that PS-NPs of different particle sizes and UV-aging states exhibit significant differences in their adsorption capacity for Cu²^+^, suggesting that they may synergistically amplify heavy metal toxicity in real-world environments (4). Due to their prevalence, stability, and high bioavailability, PS-NPs are commonly used as model particles in studies investigating the biological effects of nanoplastics (5). Both animal and cellular studies have demonstrated that nanoplastics can accumulate in organisms, leading to oxidative stress, inflammatory responses, and organ damage (6, 7). However, the effects of nanoplastics on inflammation and aging in young mammals, as well as the underlying mechanisms, remain incompletely understood.

Aging is a complex biological process characterized by a gradual decline in physiological functions over time, which is associated with the onset of various diseases (8–10). Typical markers of cellular senescence include cyclin-dependent kinase inhibitors p21^Cip1/Waf^ and p16^Ink4a^, whose high expression can lead to permanent cell cycle arrest (11). Concurrently, the reduced activity of major antioxidant enzymes such as superoxide dismutase (SOD), catalase (CAT), and glutathione peroxidase (GPX) disrupts cellular redox balance. This decline significantly aggravates oxidative damage and accelerates the aging process (12). Senescent cells further exhibit a hallmark secretory profile, termed the senescence-associated secretory phenotype (SASP) (13). The SASP constitutes a heterogeneous, multi-molecular complex comprising pro-inflammatory cytokines (e.g., interleukin-6 (IL-6), tumor necrosis factor-α (TNF-α), matrix metalloproteinases (MMPs), growth factors, and chemokines (14, 15). Critically, SASP factors propagate senescence via paracrine signaling networks: They act on neighboring cells to trigger secondary senescence, thereby driving tissue-level amplification and dissemination of the senescent phenotype (14, 16).

The p38 MAPK pathway is one of the key pathways regulating cellular senescence (17). This pathway directly promotes the expression of pro-inflammatory factors such as IL-6 and TNF-α by phosphorylating downstream transcription factors ATF2 and MEF-2C, thereby initiating and sustaining senescence-associated inflammation (18). For example, a study has shown that the phosphorylation level of p38 MAPK and the expression of pro-inflammatory cytokines, including TNF-α, IL-1β, and IL-6, are significantly upregulated in human lung tissues with increasing age (19). Notably, core components of the SASP such as IL-1β and TNF-α can further activate nuclear factor κB (NF-κB) and p38-MAPK itself, forming a positive feedback loop that continuously amplifies the inflammatory cascade and accelerates cellular senescence (20). Previous study showed that specific inhibitors blocking p38-MAPK significantly reduced SASP factor secretion levels, demonstrating this pathway’s direct regulatory role in SASP (21). The other study showed that nanoplastics activated p38 MAPK pathway by inducing oxidative stress, thereby triggering inflammatory senescence and promoting systemic aging in zebrafish models (22). Collectively, this body of evidence underscores the central role of the p38 MAPK pathway in the aging process and associated inflammatory responses, positioning it as a potential therapeutic target for anti-aging and anti-inflammatory interventions.

Oral and intestinal microbiome is an important microbial ecosystem, which plays a key role in nutrition metabolism, immune regulation and host health maintenance (23). Oral and intestinal microbiome are closely related to each other, forming an “oral and intestinal microbiome axis”, which affects each other through swallowing and immune signal transmission (24, 25). When the balance of this axis is broken, it may trigger systemic inflammatory reactions and metabolic disorders, thus accelerating the aging process (25). Polystyrene, a prevalent plastic component, can introduce nanoparticles into the oral cavity and intestinal tract through dietary intake and drinking water. This process has the potential to disrupt the oral and intestinal microbiome axis, consequently impacting overall health (26).

In summary, although existing studies have suggested that nanoplastics may induce oxidative stress and inflammatory responses, it remains unclear whether long-term oral exposure to polystyrene nanoplastics (PS-NPs) in juvenile mammals induces senescence and inflammation in the lungs and liver, whether the p38 MAPK signaling pathway plays a key role in these processes, and whether alterations in the oral-gut microbiota are associated with these phenotypes. Based on this, the present study proposes the hypothesis that PS-NPs may promote inflammatory responses and aging processes in juvenile organisms through the oral-gut microbiota axis. In this study, an 8-week-old C57BL/6 mouse model with ad libitum exposure to 1000 μg/L polystyrene (PS) nanoparticles was established to investigate the effects on aging and inflammation.

## Results

### Physicochemical characterization of PS-NPs

**Figure 1:**
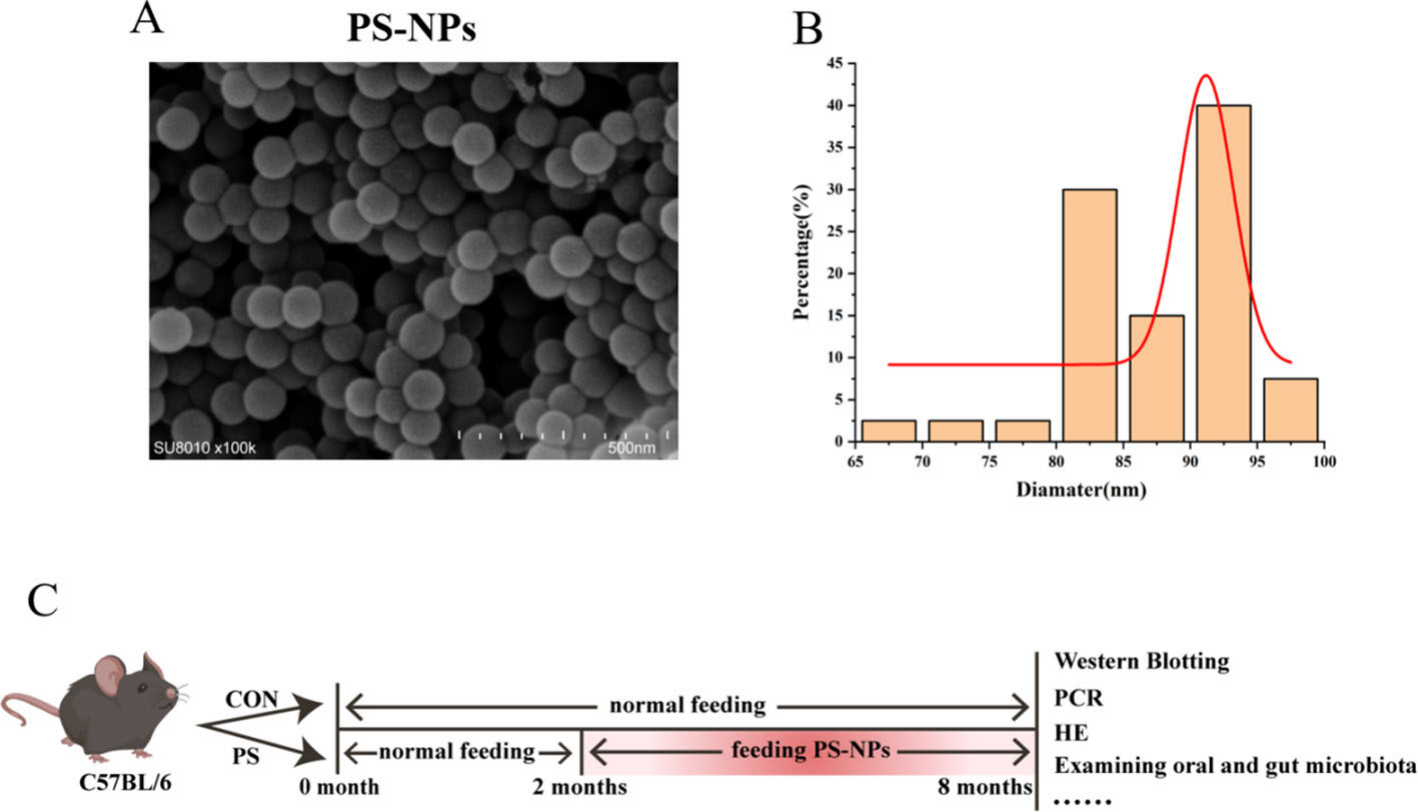
Physicochemical characterization of PS-NPs nanoparticles and overall experimental flow chart. **(A, B)** PS-NPs nanoparticle size was examined using scanning electron microscopy and particle size distribution was demonstrated; **(C)** Flow chart of the whole experiment.

The size and morphology of PS-NPs nanoparticles were determined by scanning electron microscopy, and all particles showed spherical structure and smooth surface (**Figure 1A**). Dynamic light scattering analysis of PS-NPs showed that their particle size distribution ranged from 65 to 100 nm, and PS-NPs with an average particle size of 91.2 ± 10.7 nm accounted for the largest proportion (**Figure 1B**). This characterization provides a material characterization basis for subsequent animal experiments. As illustrated in the schematic diagram of **Figure 1C**, 2-month-old C57BL/6 mice were continuously fed for 6 months via ad libitum diet before subsequent experiments were conducted.

### PS-NPs induce multi-tissue inflammatory response in young mice

**Figure 2:**
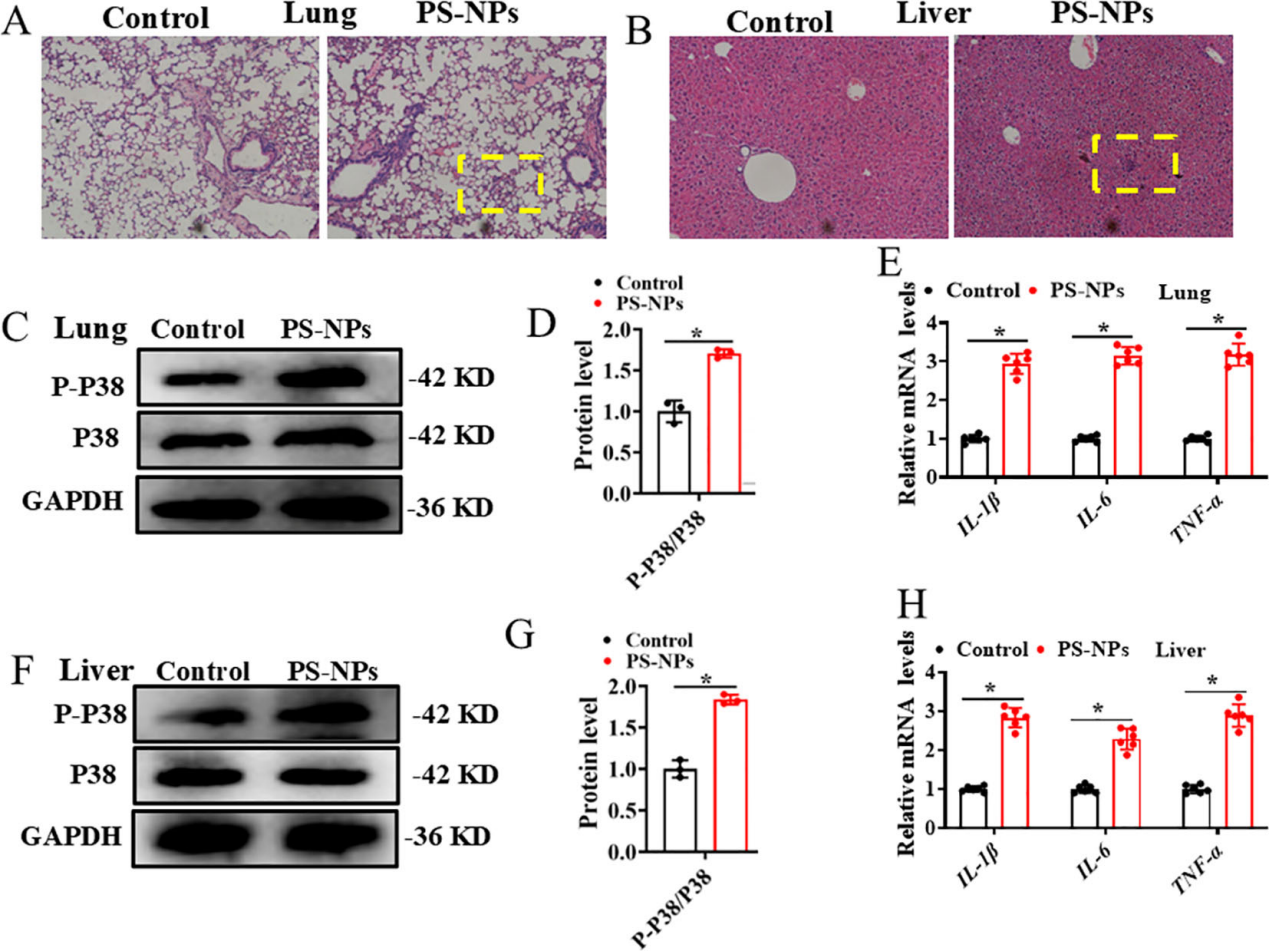
PS-NPs induce multi-tissue inflammatory response in young mice. **(A)** HE staining results of lung tissue. **(B)** HE staining results of liver tissue. **(C)** Western blot detection results of p-p38, p38, p-ERK and ERK proteins in lung tissue. **(D)** Statistical analysis of relative expression of p-p38/p38 proteins in lung tissue. **(E)** Relative mRNA levels of *IL-1β, IL-6* and *TNF-α* genes in lung tissue. **(F)** Western blot analysis of p-p38, p38, p-ERK and ERK proteins in liver tissues. **(G)** Statistical analysis of relative expression of p-p38/p38 proteins in liver tissues. **(H)** Relative mRNA levels of *IL-1β, IL-6 and TNF-α* genes in liver tissues. Data are expressed as means ± SD. (n = 6). Statistical significance was determined by unpaired Student’s t-test. *P < 0.05 vs. the control group.

Histological analysis using HE staining revealed that mice treated with PS-NPs exhibited marked inflammatory cell infiltration and disruption of alveolar architecture in lung tissue (**Figure 2A**), as well as hepatocyte edema and inflammatory cell aggregation in liver tissue (**Figure 2B**) compared to the control group. Western blot analysis demonstrated a significant elevation in the phosphorylation level of p38 (p-p38/p38) in the lung and liver tissues of the PS-NPs treatment group, indicating activation of the p38/MAPK signaling pathway. These results suggested that the PS-NPs-induced multi-tissue inflammatory response in young mice was associated with the p38/MAPK signaling pathway (**Figures 2C, D–G**). Furthermore, quantitative PCR analysis revealed a significant increase in the levels of inflammatory factors *IL-1β*, *IL-6*, and *TNF-α* in the serum of lung and liver tissues from the PS-NPs treatment group compared to the control group (**Figures 2E, H**). These findings collectively indicate that a PS-NPs free diet can trigger a multi-tissue inflammatory response in young mice.

### PS-NPs promote multi-tissue senescence in young mice

**Figure 3:**
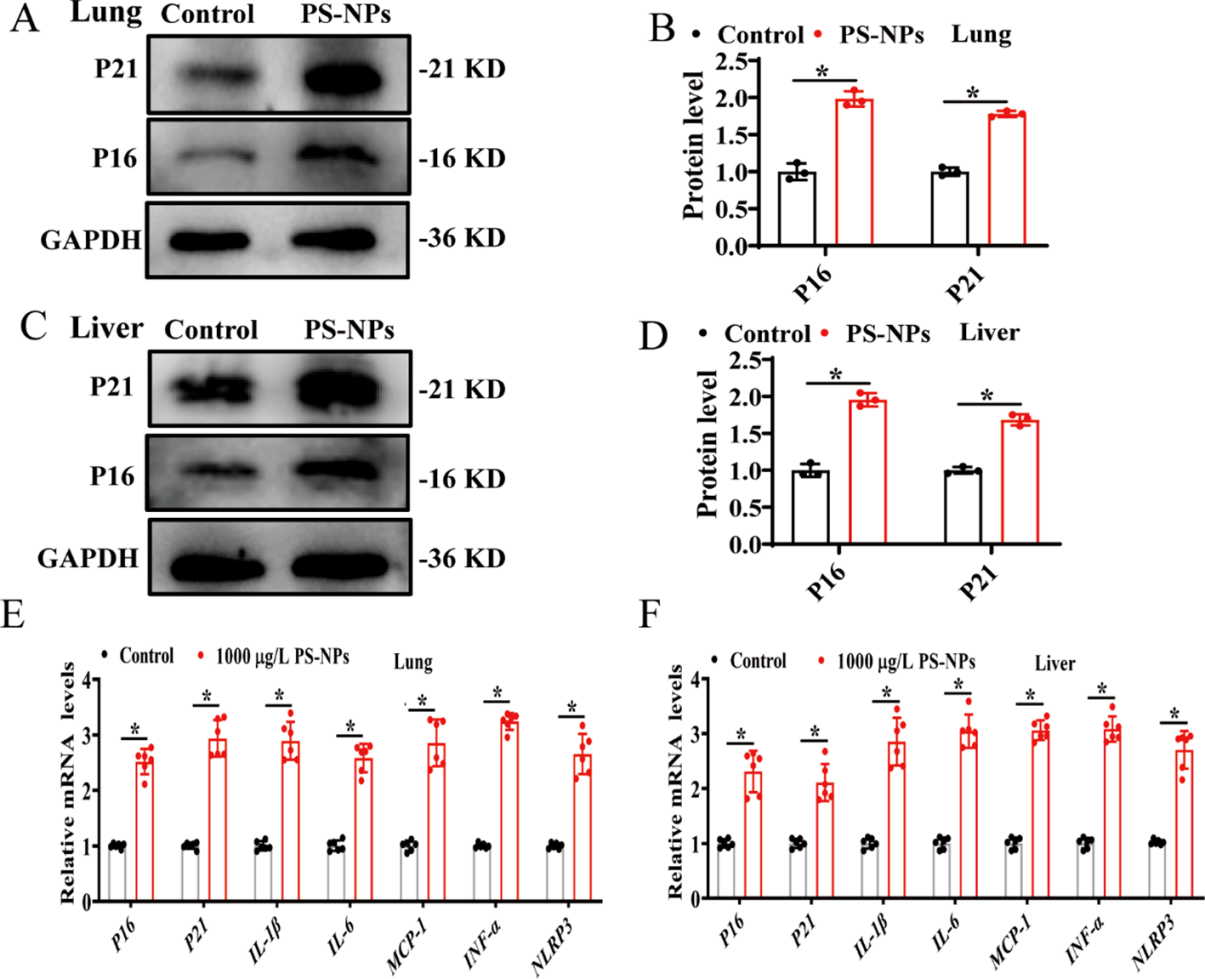
PS-NPs promote multi-tissue senescence in young mice. **(A)** Western blot analysis of P21 and P16 protein in lung tissue. **(B)** Statistical analysis of P16 and P21 protein relative expression in lung tissue. **(C)** Western blot analysis of P21 and P16 protein in liver tissue. **(D)** statistical analysis of P16 and P21 protein relative expression in liver tissue. **(E)** Relative mRNA levels of *P16, P21, IL-1β, IL-6, MCP-1, TNF-α and NLRP3* genes in lung tissue. **(F)** Relative mRNA levels of *P16, P21, IL-1β, IL-6, MCP-1, TNF-α and NLRP3* genes in liver tissue. Data are expressed as means ± SD. (n = 6). Statistical significance was determined by unpaired Student’s t-test. *P < 0.05 vs. the control group.

To assess the impact of PS-NPs on tissue aging in young mice, we analyzed the expression of SASP (13) such as cell cycle arrest core proteins p21 and p16, along with common inflammatory cytokines, in the lung and liver. Our Western blot findings revealed a significant upregulation of the senescence-related proteins p21 and p16 in the lung and liver tissues of the PS-NPs treated group compared to the control group (**Figures 3A–D**). Quantitative PCR results showed that PS-NPs treatment increased the mRNA expression levels of SASP genes such as *p21*, *p16*, *IL-1β*, *IL-6*, *MCP-1*, *INF-α*, and *NLRP3* in lung and liver tissues compared to the control group (*P* < 0.05) (**Figures 3E, F**). These findings suggest that a PS-NPs free diet can induce SASP and accelerate the aging process in young mice.

### PS-NPs induce detoxification and antioxidant dysfunction in lung and liver tissues of mice

**Figure 4:**
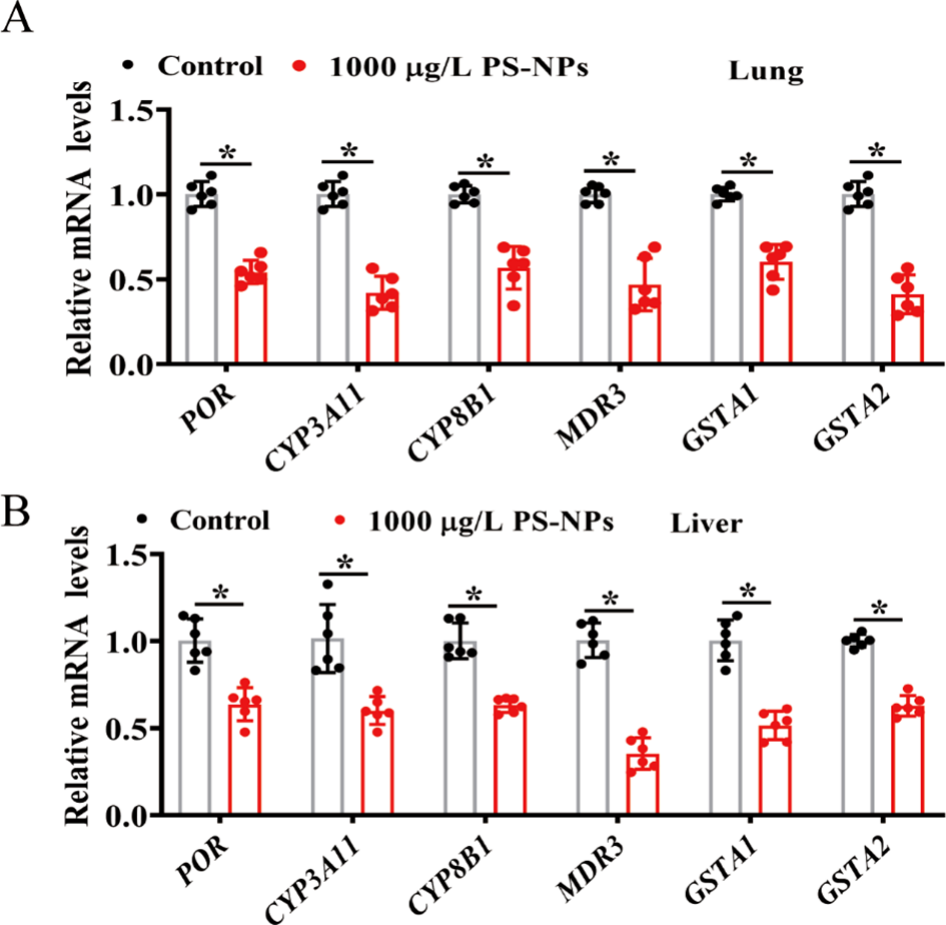
PS-NPs induce detoxification and antioxidant dysfunction in lung and liver tissues of mice. **(A)** QPCR analysis of relative mRNA expression of antioxidant genes in lung. **(B)** QPCR analysis of relative mRNA expression of antioxidant genes in liver. Data are presented as mean ± SD. (n = 6). Statistical significance was determined by unpaired Student’s t-test. *P < 0.05 vs. the control group.

To examine the impact of PS-NPs on lung and liver function, we assessed the mRNA expression levels of antioxidant genes in the respective tissues. Our findings revealed a significant reduction in the relative mRNA levels of detoxification/antioxidant-related genes in the lung tissue of the PS-NPs-treated group compared to the control group (**Figure 4A**). These included the phase I detoxification enzymes *POR* (*P* < 0.05), *CYP8B1* (*P* < 0.05), *CYP3A11* (*P* < 0.05), the phase II detoxification/antioxidant enzyme *GSTA1* (*P* < 0.05), *GSTA2* (*P* < 0.05) and the transporter *MDR3* (*P* < 0.05) (13). Similarly, exposure to PS-NPs led to a notable downregulation of these genes in liver tissue (**Figure 4B**). These results suggest that PS-NPs exposure impairs the transcription of multiple genes involved in xenobiotic metabolism and oxidative stress defense, thereby potentially compromising cellular protective capacity in mouse lung and liver tissues.

### Exposure to polystyrene nanoplastics reshapes oral microbial community structure in young mice

**Figure 5:**
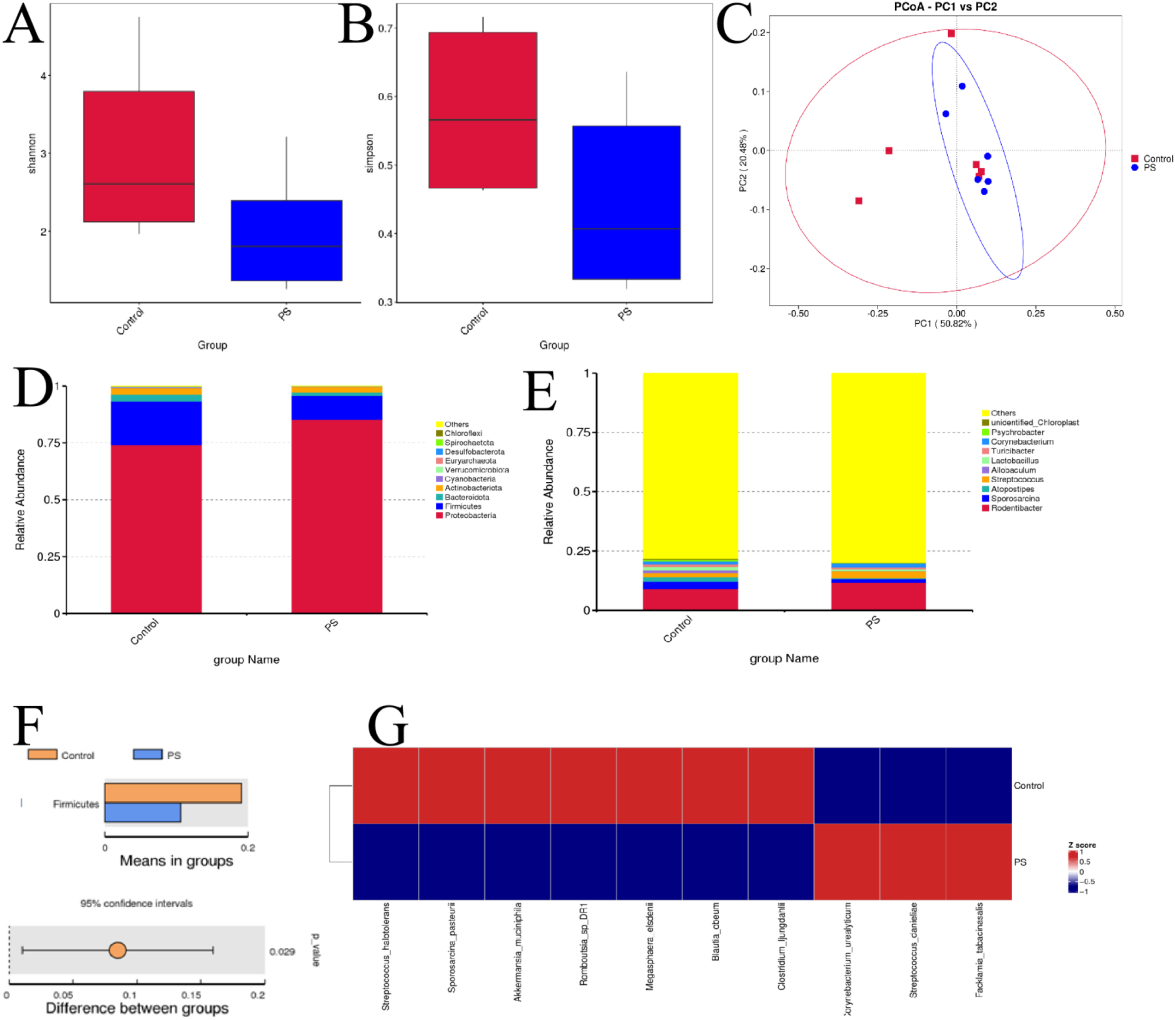
Exposure to polystyrene nanoplastics reshapes oral microbial community structure in young mice. **(A, B)** The alpha diversity indices of oral microbiota in the control and PS-NPs groups, including the Shannon index **(A)** and Simpson index **(B, C)** Principal coordinate analysis (PCoA) based on Bray-Curtis dissimilarity showing the beta diversity of oral microbiota between the control (red squares) and PS-NPs (blue circles) groups. The ellipses represent the 95% confidence intervals. **(D)** Relative abundance distribution of oral microorganisms in mice of control group (Control) and PS-NPs treatment group (PS) at phylum level. **(E)** Relative abundance distribution of oral microorganisms in mice of control group and PS-NPs treatment group at genus level. **(F)** Average relative abundance of Firmicutes in two groups and analysis of differences between groups (95% confidence interval). **(G)** Heatmap of differential genera in oral microorganisms of mice of two groups.

To assess the impact of PS-NPs on the oral microbial community structure in mice, fecal samples underwent 16S rRNA gene sequencing. Alpha diversity analysis revealed that both the Shannon index and Simpson index were significantly lower in the PS-NPs-treated group compared with the control group, indicating that PS-NPs exposure reduced the species’ richness and evenness of the oral microbial community (**Figures 5A, B**). Principal coordinate analysis (PCoA) based on Bray-Curtis distance showed clear separation of microbial community structures between the control and PS-NPs groups along the PC1 (50.82% variation explained) and PC2 (20.49% variation explained) axes, with no apparent overlap in the 95% confidence ellipses of the two groups (**Figure 5C**). These results indicated that PS-NPs exposure significantly altered the overall structure of the oral microbial community. Furthermore, analysis of the 16S rRNA sequencing data at the phylum level revealed notable compositional differences between the control and PS-NPs-treated groups. Specifically, the abundance of *Proteobacteria* significantly increased in the PS-NPs treatment group compared to the control group, whereas the abundance of *Firmicutes* exhibited a significant decrease (**Figure 5D**). Subsequent analysis at the genus level unveiled significant alterations in the composition of oral microbial genera in the PS-NPs treated groups. This included a marked reduction in beneficial bacteria such as *Lactobacillus* and *Ackermannia*, alongside a considerable increase in other bacterial genera like *Streptococcus* and *Corynebacterium* in the PS-NPs treated groups as opposed to the control groups (**Figures 5E, G**). *Firmicutes* play a crucial role in various physiological processes within the oral. A reduction in their abundance can impact both the metabolic functions and immune regulation of the oral. Statistical analysis focusing on *Firmicutes* revealed a significant difference in the average relative abundance between the PS-NPs treatment group and the control group (*P* < 0.05). The 95% confidence interval and inter-group difference analysis indicated a down-regulation in *Firmicutes* abundance (**Figure 5F**). These results suggest that exposure to PS-NPs could disrupt oral metabolic functions and immune regulation, consequently influencing the overall health of mice. Heatmap showed significant alterations in the relative abundance of multiple bacterial genera in the oral of mice following exposure to PS-NPs (**Figure 5G**). These results indicate that PS-NPs have the potential to disturb the balance of the oral microecosystem by restructuring the composition and structure of the intestinal microbiome. These changes may play a role in mediating the health effects on the host induced by nanoplastic exposure. The findings suggest that a PS-NPs free diet can induce notable changes in the structure of the oral microbial community in mice. These alterations may have adverse effects on the balance of the oral microecosystem, consequently impacting the overall health of the mice.

### Exposure to polystyrene nanoplastics reshapes intestinal microbial community structure in young mice

**Figure 6:**
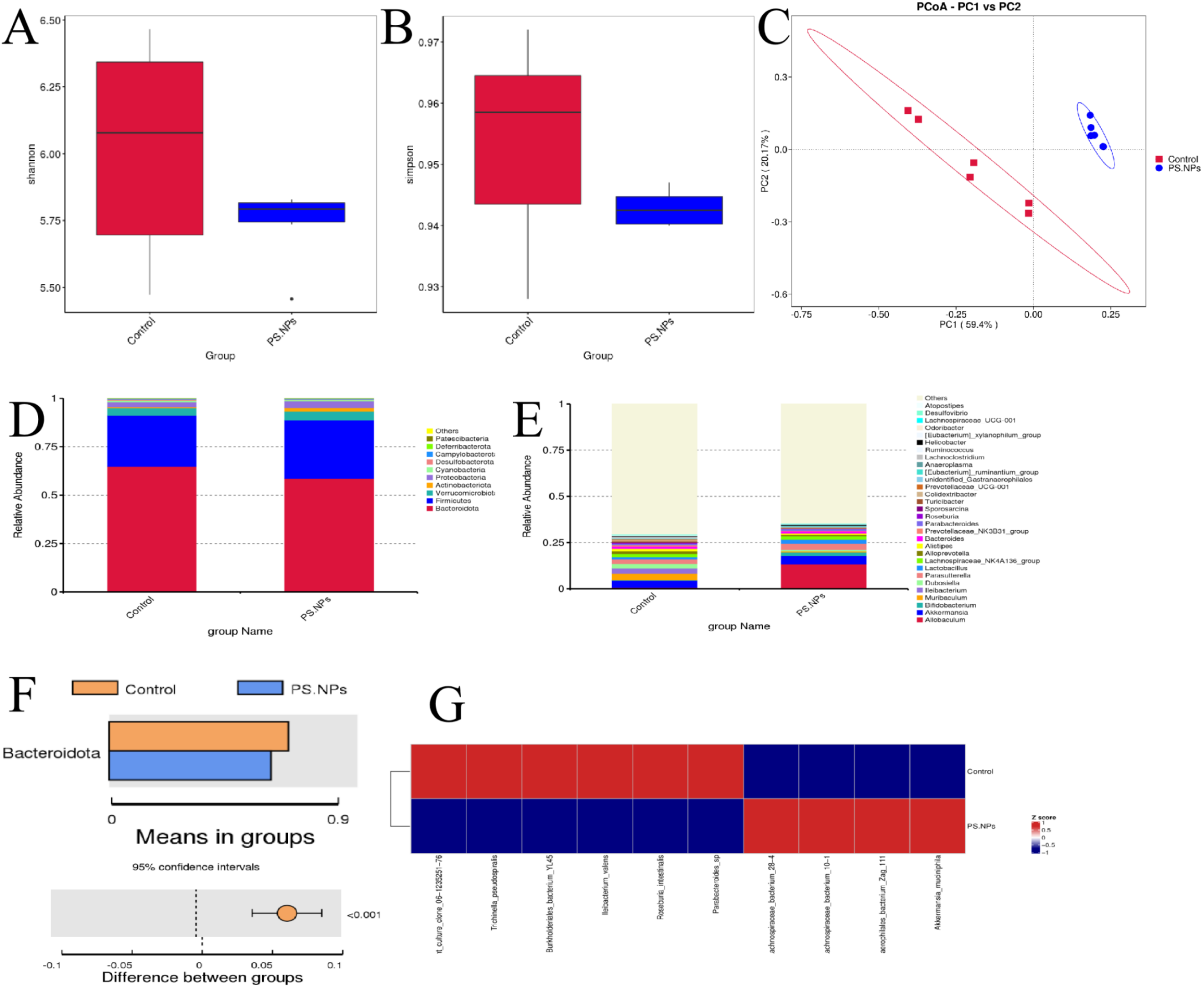
Exposure to polystyrene nanoplastics reshapes intestinal microbial community structure in young mice. **(A, B)** The alpha diversity indices of gut microbiota in the control and PS-NPs groups, including the Shannon index **(A)** and Simpson index **(B, C)** Principal coordinate analysis (PCoA) based on Bray-Curtis dissimilarity showing the beta diversity of gut microbiota between the control (red squares) and PS-NPs (blue circles) groups. The ellipses represent the 95% confidence intervals. **(D)** Relative abundance distribution of intestinal microorganisms in control group and PS-NPs treated group at phylum level. **(E)** Relative abundance distribution of intestinal microorganisms in control group and PS-NPs treated group at genus level. **(F)** Average relative abundance of Bacteroidota in two groups and analysis of differences between groups (95% confidence interval). **(G)** Heatmap of differential genera in intestinal microorganisms in two groups.

Consistent with the alterations observed in the oral microbial community, the gut microbiota also underwent profound structural remodeling following PS-NPs exposure. Alpha diversity analysis revealed that both the Shannon index and Simpson index were significantly lower in the PS-NPs-treated group relative to the control group, indicating that PS-NPs exposure reduced the species diversity and evenness of the gut microbial community (**Figures 6A, B**). Principal coordinate analysis (PCoA) based on Bray-Curtis distances revealed a clear separation of gut microbial communities between the control and PS-NPs-treated groups along the PC1 (59.4% variation explained) and PC2 (20.17% variation explained) axes, with no overlap in the 95% confidence ellipses of the two groups. These results indicate that PS-NPs exposure significantly altered the overall structure of the gut microbial community (**Figure 6C**). Analysis at the phylum level revealed differences in the relative abundance distribution of intestinal microflora in the PS-NPs treated group, with a slight downregulation of *Bacteroidota* compared to the control group (**Figure 6D**). Examination at the genus level indicated significant differences in the composition of intestinal microbial genera between the PS-NPs treated and control groups, with alterations in the relative abundance of multiple genera (**Figure 6E**). Specifically focusing on *Bacteroidota* (**Figure 6F**), the mean relative abundance of this phylum was slightly reduced in the PS-NPs treated group compared to the controls. Analysis of different bacterial genera (**Figure 6G**) demonstrated significant inter-group differences in the relative abundance of *Trichotherium*, *Bacteroides*, and other bacterial genera between the control and PS-NPs treatment groups, suggesting that PS-NPs can modify the composition and structure of the intestinal microbiome, influencing intestinal microecology. These changes may play a role in mediating the intestinal health effects associated with nanoplastic exposure.

## Discussion

Administered PS-NPs via intraperitoneal injection exacerbated lipopolysaccharide (LPS)-induced duodenal inflammation and increased intestinal permeability in mice through ROS-driven NF-κB/NLRP3 pathways (27). Administered PS-NPs via oral gavage induced hematopoietic damage through crosstalk involving the gut microbiota, metabolites, and cytokines (28). Early-life exposure to polystyrene microplastics and nanoplastics via the drinking water route disrupts metabolic homeostasis and gut microbiota in juvenile mice in a size-dependent manner (29). In this study, we innovatively employ a 1000 μg/L PS-NPs free-diet exposure model, which simulates environmentally relevant low-dose chronic exposure scenarios and more closely approximates real-world biological exposure conditions. Meanwhile, this study focuses on young mice, further highlighting the impact of nanoplastics on individuals who have not yet entered the natural aging phase, thereby offering a new perspective for early health risk assessment of nanoplastics.

Oxidative stress is a key driver in the ageing process, with excessive reactive oxygen species causing damage to biomolecules such as DNA, proteins, and lipids, thereby disrupting cellular function (30). Previous studies have demonstrated that nanoplastics can participate in ageing regulation by inducing oxidative stress. For instance, polystyrene nanoplastics elevate intracellular ROS levels and reduce antioxidant enzyme activity (27). Monocyte chemotactic protein-1 (MCP-1) exhibits a close association with oxidative stress (31). In this study, exposure to PS-NPs led to upregulation of MCP-1 gene expression in the liver and lung tissues of young mice, further corroborating the mechanism whereby nanoplastics promote ageing via oxidative stress. In addition, our work revealed that PS-NPs decreased the expression of antioxidant genes, including *POR*, *CYP3A11*, *CYP8B1*, *MDR3*, *GSTA1*, and *GSTA2*. The enzymes encoded by these genes play crucial roles in ROS scavenging and detoxification processes. Their downregulation further impairs cellular antioxidant defence capabilities (32, 33). For example, *CYP3A11* and *CYP8B1* are involved in xenobiotic metabolism and maintaining redox balance, with their suppressed expression potentially exacerbating oxidative damage (34). Collectively, these findings indicate that PS-NPs accelerate ageing by enhancing oxidative stress through weakening cellular antioxidant defence systems.

The p38 MAPK pathway, as a key molecular pathway associated with ageing, regulates the transcription and release of pro-inflammatory factors through a cascade of phosphorylation events upon activation, thereby mediating chronic inflammatory responses (35). Previous studies have demonstrated that PS-NPs induce dysregulation of glucose and lipid metabolism in mice via the NF-κB and MAPK pathways (36). Furthermore, PS-NPs exacerbate colitis through the p38 MAPK pathway (37). In this study, we observed significant activation of the p38 MAPK pathway following PS-NP exposure, accompanied by increased secretion of SASP factors. This finding aligns with prior conclusions, indicating that PS-NPs trigger an ‘oxidative stress-inflammation’ cascade by activating the p38 MAPK pathway, ultimately accelerating the ageing process. Furthermore, our study demonstrates that prolonged exposure to PS-NPs in young mice significantly elevates serum levels of pro-inflammatory cytokines IL-1β, IL-6, and TNF-α. This phenomenon aligns with the mechanism whereby the SASP drives cellular ageing, consistent with findings from multiple rodent model studies. It indicates that polystyrene nanoplastic exposure can induce systemic inflammatory responses (38).

The oral-gut microbial axis plays a crucial role in maintaining host health and modulating the aging process (39). Under physiological conditions, dominant phyla such as *Bacteroidetes* and *Firmicutes* suppress inflammatory responses through microbial metabolites (40). Conversely, microbial dysbiosis characterized by an increase in pro-inflammatory bacterial genera is positively correlated with the activation of SASP, a hallmark of aging (41, 42). This study demonstrates that PS-NPs exposure disrupts the composition and function of the oral-gut microbiota. Specifically, PS-NPs reduce the abundance of beneficial bacteria (e.g., *Lactobacillus*, *Ackermannia*) while promoting the proliferation of pro-inflammatory taxa. These structural alterations align with observed phenotypes of enhanced systemic inflammation and accelerated aging in mice. Mechanistically, PS-NPs likely compromise the homeostasis of the microbial axis, thereby disrupting host-microbiota equilibrium. This disruption may trigger systemic inflammation via increased gut permeability and endotoxin translocation (e.g., lipopolysaccharide, LPS), which activates NF-κB signaling and elevates inflammatory cytokines such as TNF-α and IL-6. Concurrently, dysbiosis-induced SASP exacerbates cellular senescence.

The present study has several limitations. First, only a single dose (1000 μg/L) was used, which precludes the establishment of a dose-response relationship. Future studies should include at least three dose groups (e.g., 100, 500, and 1000 μg/L) to systematically evaluate dose–response effects. Second, antibiotic depletion or fecal microbiota transplantation (FMT) experiments were not performed; therefore, the association between the microbiota and host phenotypes remains correlative, and causality cannot be established. Future studies are needed to directly validate causality using FMT and antibiotic intervention. Third, a p38 MAPK inhibitor (e.g., SB203580) was not administered *in vivo*, and thus the necessity of this pathway has not been demonstrated. Future studies should incorporate inhibitor intervention in short-term exposure models to validate the role of p38 MAPK. Fourth, reactive oxygen species (ROS) were not directly measured, which limits direct support for the involvement of oxidative stress. Subsequent studies should routinely measure ROS in fresh tissues or cells. Fifth, direct evidence of *in vivo* PS-NP ingestion (e.g., via fluorescence labeling or Py-GC/MS) is lacking, making it impossible to confirm whether the particles are truly distributed to target organs. Sixth, it should be noted that this study did not provide real-time stability or zeta potential data of PS-NPs in the exposure medium, which may limit the interpretation of biological effects associated with particle dispersion state and surface charge. These limitations do not diminish the correlative value of our findings, but causal relationships and mechanistic validation need to be addressed in future studies. We sincerely acknowledge these shortcomings and will address them in our subsequent work.

## Conclusion

**Figure 7:**
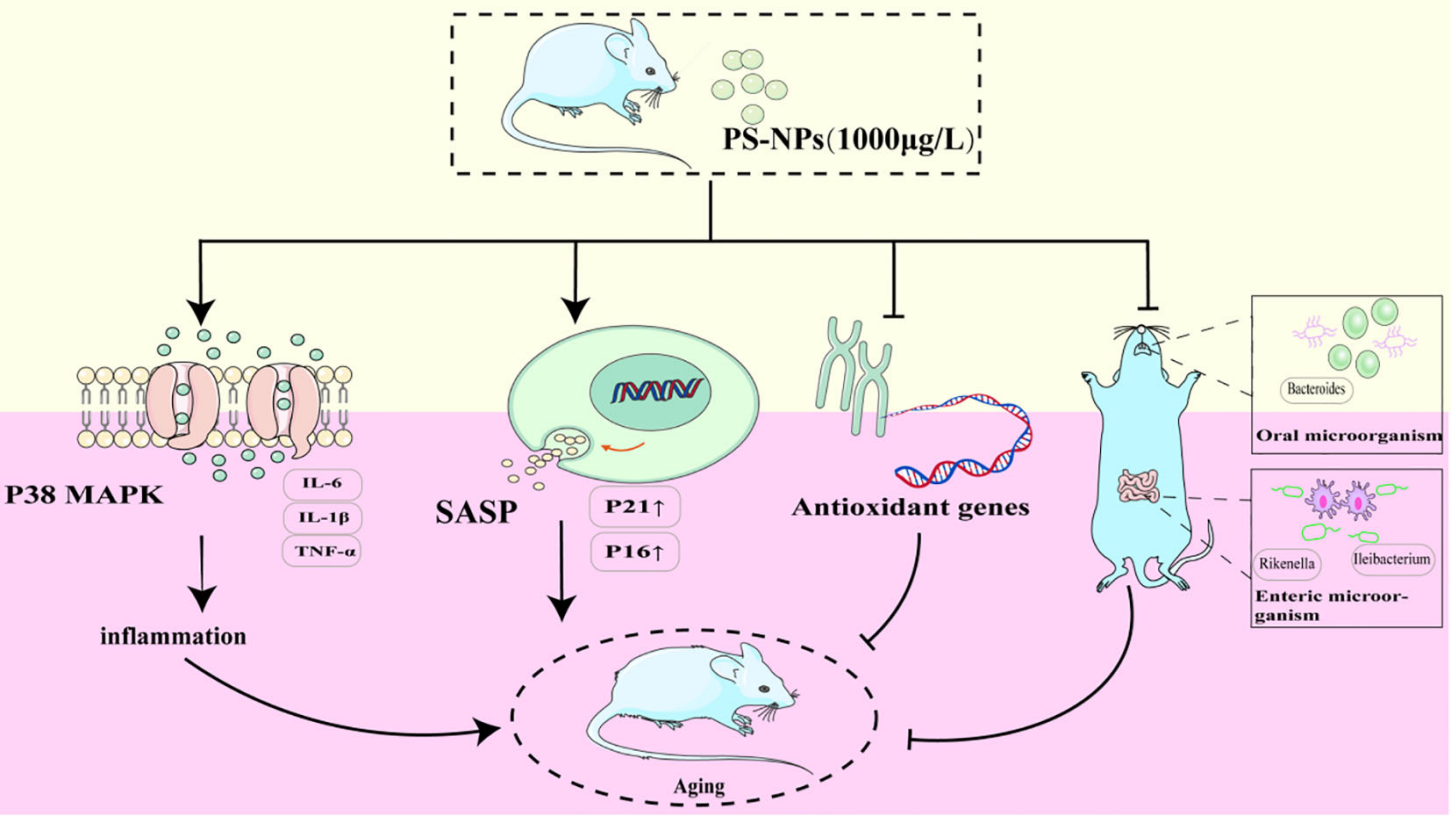
Schematic diagram illustrating the mechanisms by which polystyrene nanoparticles (PS - NPs, 1000 μg/L) induce aging in mice.

In summary, this study confirms that exposure to PS-NPs activates the p38 MAPK pathway, leading to elevated levels of inflammatory cytokines, upregulation of cellular senescence markers, and downregulation of antioxidant gene expression. Concurrently, it disrupts the oral-gut microbiome axis homeostasis, ultimately accelerating the ageing process in mice (**Figure 7**). These findings elucidate the toxicological mechanisms of nanoplastics at molecular and microbiological levels, providing novel theoretical foundations for assessing their potential health risks in adolescents. Future research should further investigate interactions between nanoplastics and other environmental factors to furnish more comprehensive scientific support for developing relevant public health strategies.

## Materials and methods

### Chemicals

PS-NPs nanoparticles used in this study were purchased from Macklin (Shanghai, China). Nanoparticles were examined for particle size and morphology using scanning electron microscopy.

### Animals

7-week-old male C57BL/6 mice were reared in the Animal Center of Zunyi Medical University. Mice were acclimated for one week prior to the experiment. They were then randomly assigned to the control group and the PS-NPs exposure group using a random number table method, with six mice per group. Specifically, after sorting the mice by body weight, random numbers were generated using Excel, and the animals were allocated according to the ascending order of the random numbers. Control mice were fed ad libitum for 6 months, and PS-NPS treated mice were fed ad libitum for water at 1000 μg/L PS-NPS for 6 months to mimic natural human microplastic intake. During the experiment, the personnel responsible for animal housing and oral gavage were separated from those performing sample detection and data analysis. All tissue samples were labeled with unique codes after collection. The investigators performing the assays (e.g., Western blot, qPCR, histopathological analysis) and data analysis were blinded to the group allocation until all data had been completely processed and the codes were broken. After the experiment, the mice were killed by cervical vertebrae for follow-up experiments. ARRIVE guidelines Compliance:All methods are reported in accordance with ARRIVE guidelines. All mouse studies were carried out under standard conditions and in accordance with Zunyi Medical University Animal Care Committee (ZMU21-2409-008) guidelines. All animals were obtained from Zunyi Medical University.

### Pathological analysis

Mouse liver and lung tissues were fixed in 10% formalin for 24 hours, followed by serial dehydration with ethanol and xylene before paraffin embedding. Paraffin-embedded specimens were sectioned into 8μm-thick tissue sections and subjected to haematoxylin and eosin (H & E) staining. Stained tissue sections were examined under an optical microscope.

### Western blot

Protein samples from lungs and liver were subjected to SDS-PAGE and electroporated onto PVDF membranes. Subsequently, the membrane was blocked with flash blocking solution for 15min at room temperature and then washed with PBST solution for 10min, repeated 3 times. Membranes were then incubated overnight with primary antibody at 4 °C. The membrane was then washed in PBST solution for an additional 10min, repeated three times, and incubated with secondary anti-rabbit or anti-mouse antibodies for 2 hours at room temperature. Subsequently, the luminescent substrate working solution was applied to the membrane and the protein bands were visualized by exposure using BIO-RAD ChemiDoc imaging system followed by quantification using imageJ.

### Quantitative real-time fluorescence quantitative PCR

Total RNA was extracted from liver and lung using Vazyme Trizol Total RNA Extraction Kit. After RNA concentration was determined, RNA was reverse transcribed into cDNA using HiScript^®^ II Q RT SuperMix for qPCR reverse transcription kit. The real-time quantitative qPCR reaction system was then prepared according to ChamQ Universal SYBR qPCR Premix Operating Instructions, and the real-time quantitative qPCR reaction was performed according to the recommended protocol. mRNA expression was calculated by 2^-△△Ct^ method with GAPDH as internal reference. The specific primer sequences are as follows:.

*p21* primers:.

*p21*-F: CGATGGAACTTCGACTTTGTCA.

*p21*-R: GCACAAGGGTACAAGACAGTG.

*p16* primers:.

*p16*-F: CCCAACGCCCCGAACT.

*p16*-R: GCAGAAGAGCTGCTACGTGAA.

*IL-6* primers:

*IL-6*-F: CTCTGCAAGAGACTTCCATCCAGT.

*IL-6*-R: GAAGTAGGGAAGGCCGTGG.

*IL-1β* primers:.

*IL-1β*-F: GTGGCTGTGGAGAAGCTGTG.

*IL-1β*-R: GAAGGTCCACGGGAAAGACAC.

*TNF-α* primers:.

*TNF-α*-F: AGGGTCTGGGCCATAGAACT.

*TNF-α*-R: CCACCACGCTCTTCTGTCTAC.

*MCP1* primers:.

*MCP1*-F: TTAAAAACCTGGATCGGAACCAA.

*MCP1*-R: GCATTAGCTTCAGATTTACGGGT.

*NLRP3* primers:.

*NLRP3*-F: *AGCCAGAGTGGAATGACACG*.

*NLRP3*-R: *GCGCGTTCCTGTCCTTGATA*.

*CYP3A11* primers:.

*CYP3A11*-F: GTGCTCCTAGCAATCAGCTT.

*CYP3A11*-R: CAGTGCCTAAAAATGGCAGAGG.

*CYP8B1* primers:.

*CYP8B1*-F: GGACAGCCTATCCTTGGTGA.

*CYP8B1*-R: GACGGAACTTCCTGAACAGC.

*POR* primers:.

*POR*-F: CGGTGTTGCTGTTGGTATTG.

*POR*-R: TGTCCTGGTTGTCCTGGTT.

*GSTA1* primers:.

*GSTA1*-F: CCCCTTTCCCTCTGCTGAAG.

*GSTA1*-R: TGCAGCTTCACTGAATCTTGAAAG.

*GSTA2* primers:.

*GSTA2*-F: GCAGGGGTGGAGTTTGAAGA.

*GSTA2*-R: AGAATGGCTCTGGTCTGCAC.

*MDR3* primers:.

*MDR3*-F: ATGGCCCTACTTTGTCGTGG.

*MDR3*-R: CTGCTTCACTGCATCATCGC.

### 16S rRNA gene amplicon sequencing of oral and gut microbiota in mice

This study employed 16S ribosomal RNA (rRNA) gene sequencing analysis on fecal samples and oral samples obtained from mice. First, genomic DNA was extracted from microbial samples in mouse faeces and oral cavities. Subsequently, the V4 region of the 16S rRNA gene was amplified using primers (515F: GTGCCAGCMGCCGCGGTAA, 806R: GGACTACHVGGGTWTCTAAT). Following successful amplification and quality control of DNA samples, PCR products were pooled and purified. Library preparation was completed through terminal repair, A-tailing, sequencing adapter ligation, and further purification. Libraries were then subjected to paired-end sequencing on an Illumina platform. Following read assembly and filtering, amplicon sequence variants (ASVs) were denoised. The resulting valid data underwent species annotation and abundance analysis to reveal the species composition of the samples. Further analysis of α-diversity and β-diversity was conducted to assess differences in community structure between samples. with dry ice for 16S rRNA gene sequencing oral microorganisms. The raw sequencing data have been submitted to the NCBI Gene Expression Omnibus (GEO) under the accession number PRJNA1268956.

### Statistical analysis

All data are expressed as a standard deviation (SD) ± mean and all results are independently repeated at least 3 times. GraphPad Prism 9.0 software was used for statistical analysis. One-way ANOVA and t-test were used to analyze data differences. *P* < 0.05 was considered statistically significant.

## Data Availability

The datasets presented in this study can be found in online repositories. The names of the repository/repositories and accession number(s) can be found below: https://www.ncbi.nlm.nih.gov/geo/, PRJNA1268956.
